# Exploiting Silica-Binding and Silica-Forming Proteins as Versatile Tools for One-Step Enzyme Immobilization on Siliceous Materials

**DOI:** 10.3390/ijms26031304

**Published:** 2025-02-03

**Authors:** Gyun Taek Lim, Byung Hoon Jo

**Affiliations:** 1 Division of Applied Life Science (BK21 Four) and Anti-Aging Bio Cell Factory Regional Leading Research Center (ABC-RLRC), Gyeongsang National University, Jinju 52828, Republic of Korea; lim06270927@gnu.ac.kr; 2 Division of Life Science and Research Institute of Life Science, Gyeongsang National University, Jinju 52828, Republic of Korea

**Keywords:** enzyme immobilization, silica-binding peptide, silica-forming peptide, biomimetic silica, fusion protein

## Abstract

Enzyme immobilization has emerged as an essential technique in industrial applications of enzymes. Silica (SiO_2_) serves as a prominent support material for enzyme immobilization. Recent advancements have led to the development of various silica-binding proteins (SBPs) and silica-forming proteins (SFPs) that are invaluable tools in immobilizing enzymes on siliceous materials in a fast and simple manner. SBPs facilitate the immobilization of enzymes with controlled orientation on silica surfaces, while SFPs enable the biomimetic synthesis and encapsulation of enzymes within silica particles. In this review, we explore recent advances in the use of SBPs and SFPs in enzyme applications. We provide a comprehensive overview of their mechanisms and sequence characteristics relevant to enzyme immobilization. Additionally, we summarize the recombinant production and immobilization procedures for enzymes with SBPs or SFPs. We then categorize the available SBPs and SFPs into naturally occurring and artificially engineered types, presenting recent examples that demonstrate their utilization in enzyme immobilization. Our review highlights the strengths and limitations of various SBPs and SFPs and sheds light on future directions for the development of tailor-made biocatalytic silica.

## 1. Introduction

Enzyme-catalyzed processes are becoming more important in large-scale industries such as the environment, pharmaceutical, and food industries. However, the use of free enzymes often has limitations, such as low stability and difficulties in recovery and reuse, thereby increasing cost. Enzyme immobilization can improve the stability of enzymes under reaction conditions such as high acidity/alkalinity, high temperatures, or high contents of organic solvents. It can also facilitate the separation and reuse of enzymes from reaction solutions [1].

Various support materials have been utilized for enzyme immobilization, including inorganic materials, organic polymers, and biological scaffolds [1,2,3]. Among them, silica (SiO_2_) is a low-cost and earth-abundant matrix that is one of the most widely used supports for enzyme immobilization. Because of its high surface area, mechanical stability, biocompatibility, and chemical resistance, silica can be used not only as a matrix for biocatalysts and biosensors but also as a carrier for drug delivery systems and cosmetic additives [4,5].

**Figure 1:**
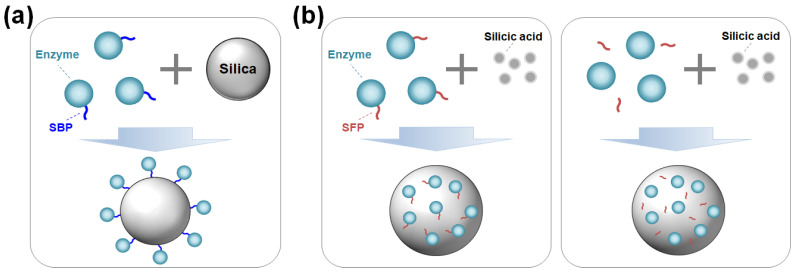
(**a**) Oriented enzyme immobilization on silica surface by a silica-binding peptide/protein (SBP) fusion. (**b**) Enzyme encapsulation within silica particles using a silica-forming peptide/protein (SFP) either in a genetically fused form (**left**) or in a physically mixed form (**right**) with the SFP.

Enzymes can be immobilized on silica surfaces through either covalent bonding or adsorption. The covalent bonding method ensures irreversible and strong immobilization but requires silica functionalization and a chemical reagent to crosslink the enzyme with the modified silica [6]. In contrast, immobilization by adsorption via noncovalent bonds offers a simple, fast, and cost-effective alternative. However, the interaction between the enzyme and the support is generally nonspecific and weak, which can negatively affect enzyme activity or stability [1]. Moreover, not all enzymes can be effectively immobilized onto silica through simple adsorption. Recent developments of silica-binding proteins/peptides (SBPs) have provided universal tools to address the limitations of traditional immobilization methods. By serving as fusion tags with a strong affinity for silica, SBPs can anchor target enzymes strongly on unmodified silica with controlled orientation (Figure 1a) [7,8].

Advances in material science combined with biochemistry have driven the development of protein- and peptide-directed syntheses of inorganic materials [5,9,10]. Inspired by the biosilica of marine diatoms and sponges, silica-forming proteins/peptides (SFPs) have been discovered and utilized for the innovative synthesis of silica nanomaterials. SFPs can synthesize silica from silica precursors (e.g., orthosilicic acid) by polymerizing these precursors and inducing the aggregation of silica particles [11]. By exploiting this biomimetic process for enzyme immobilization, target enzymes—either in the form of being genetically fused or physically blended with an SFP—can be encapsulated in situ within the synthesized silica particles under neutral pH and room temperature conditions over a short amount of time (Figure 1b).

**Figure 2:**
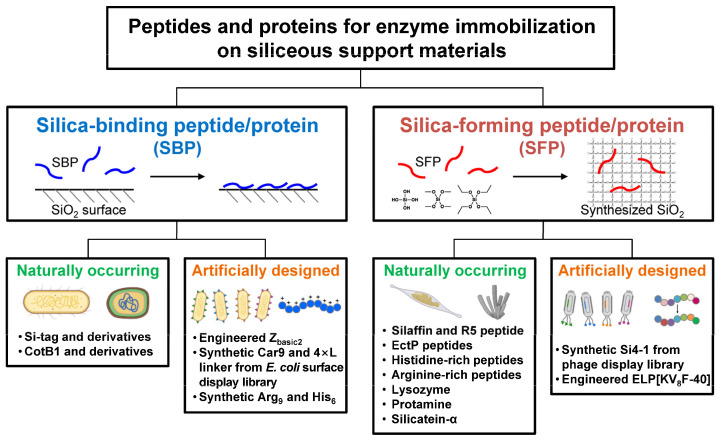
Schematic representation of silica-binding and silica-forming peptides/proteins, each categorized into naturally occurring and artificially engineered types.

In general, though with some exceptions, both SBPs and SFPs are characterized by having a high isoelectric point (pI) value with a high content of positively charged amino acids (Lys, Arg, His). SFPs are generally considered to be SBPs, but not vice versa [12], implying that a more elaborate sequence design is required to confer silica-forming functionality. In this review, SBPs and SFPs relevant to enzyme technologies are categorized into naturally occurring and artificially engineered ones, and their distinct features, including the working mechanisms, are summarized (Figure 2). A naturally occurring protein refers to a protein or a portion of a protein with a native, unmodified stretch of amino acid sequence. Conversely, an artificially engineered protein may be a variant whose sequence has been randomly or rationally mutated from its native form, or it may be a synthetic protein with an entirely new sequence with no previously identified homologues. This review may guide us in choosing proper SBPs or SFPs for effectively immobilizing enzymes on siliceous materials.

## 2. Silica-Binding Proteins/Peptides (SBPs)

### 2.1. How SBPs Work

**Figure 3:**
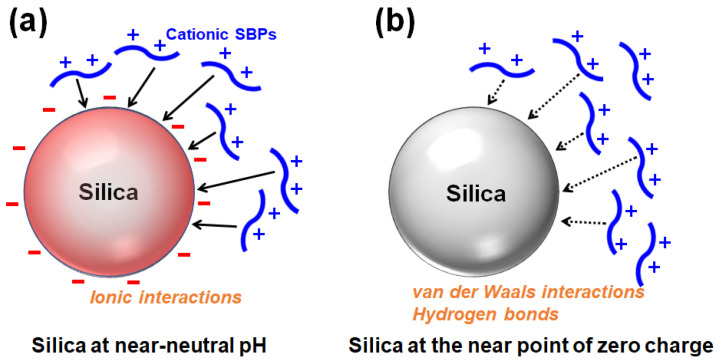
Adsorption of positively charged silica-binding peptides/proteins onto silica surface (**a**) primarily via ionic interactions at near-neutral pH or (**b**) via van der Waals interactions and hydrogen bonds at the near point of zero charge (redrawn from reference [14]).

The silanol groups (Si-OH) are exposed on the surface of silica, and the silanols (p*K*_a_ ≒ 7) are deprotonated to be silanolates (Si-O^−^) depending on the pH [13]. Proteins or peptides rich in positively charged residues can bind to and be adsorbed onto the negatively charged surface of silica via ionic interactions (Figure 3a). Accordingly, cationic SBPs with a high pI can bind to the silica surface via ionic interactions. When the pH is lowered and the silica surface approaches the point of zero charge (pzc), cationic SBPs can be adsorbed primarily via nonionic interactions such as van der Waals interactions and hydrogen bonds (Figure 3b) [14,15]. Because enzymes are generally expected to be immobilized and utilized near neutral pH, SBPs widely used as a strong binder for enzyme immobilization are mostly cationic. The kinetics and thermodynamics of SBPs binding to silica in a monolayer can be described using the Langmuir adsorption model [8,16,17], while the Freundlich model is applicable for multilayer adsorption [14].

In addition, SBPs that are mainly composed of non-polar amino acids, such as the S2 peptide with the sequence AFILPTG, prefer hydrophobic interactions with the silica surface [14]. These interactions are strengthened when the silica surface gets closer to the pzc. On the contrary, when the silica surface is more negatively charged, a higher concentration is required for the adsorption of those hydrophobic SBPs to occur [14,15].

Collectively, the contribution of each interaction to the binding of an SBP to the silica surface may vary depending on the pH condition, pI of the SBP, and both the composition and arrangement of amino acids of the SBP [15,18]. The amino acid sequences and the pI values of various SBPs are summarized in Table 1.

### 2.2. Production and One-Step Immobilization of SBP-Fused Enzyme

**Figure 4:**
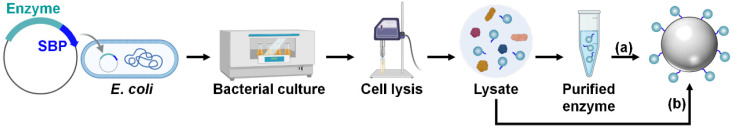
Recombinant production and immobilization of SBP-fused enzyme. One-step enzyme immobilization is achieved by mixing either (**a**) purified enzyme solution or (**b**) cell lysate with a silica matrix.

For SBP-mediated immobilization, enzymes should be produced as fusion proteins tagged with an SBP at the N-terminus, C-terminus, or both to improve the immobilization efficiency (Figure 4) [26]. For the cost-effective production of large quantities of enzymes, their high-level recombinant expression is necessary, which is typically achieved in *Escherichia coli* strains such as BL21(DE3) using strong promoters such as T7 [27]. Once the enzymes are successfully overproduced, the bacterial cells are lysed to release intracellular proteins, including the fusion enzyme. From this cell lysate, the SBP-fused enzyme is then purified, often via one-step affinity purification using an affinity tag such as the hexahistidine (His_6_) tag, or via multiple purification steps to enhance purity. The subsequent one-step enzyme immobilization is performed by mixing the purified enzyme solution with a silica material under ambient aqueous conditions (Figure 4a). Alternatively, for a more straightforward and efficient process, the SBP-fused enzyme can be selectively and directly immobilized by simply mixing the cell lysate with the silica matrix, bypassing the enzyme purification steps (Figure 4b) [26].

### 2.3. Naturally Occurring SBPs

#### 2.3.1. Si-Tag and Its Derivatives

The Si-tag is the other name of *E. coli* L2, a ribosomal protein with 273 amino acids. This SBP has been discovered by bioprospecting *E. coli* proteins that can strongly bind to silica under 1 M NaCl conditions where ionic interactions are generally suppressed [19]. The binding is strong with a dissociation constant (*K*_d_) of 0.7 nM and is virtually irreversible without salt and detergent. The positively charged, intrinsically disordered N-terminal (1–60) and C-terminal (203–273) domains are cooperatively required for strong silica binding, while the central domain (61–202) is not responsible for the silica-binding ability of the Si-tag [19]. Accordingly, a shortened version (L2NC) of the Si-tag has been created by removing the central domain and directly fusing the N-terminal region with the C-terminal region [20]. The Si-tag contains 63 positively charged residues, crucial for strongly binding to silica via ionic interactions. In addition, the efficient binding of the Si-tag can occur even under high-salt and denaturing conditions. This can be explained by conformational adaptation of the intrinsically disordered regions of the Si-tag at the binding interface, by which not only ionic interactions between cationic side chains and silanol groups but also hydrophobic interactions between apolar side chains and hydrophobic siloxane sites are optimized [28].

By utilizing the L2NC tag, Kim et al. immobilized carbonic anhydrase (CA) on diatomite for carbon dioxide (CO_2_) capture applications [20]. The thermal stability of the enzyme was improved by the self-immobilization of the enzyme in multilayers at a high packing density. Intriguingly, the immobilization system showed a phenomenon of activity−stability trade-off according to the amount of enzyme loading, providing a unique model for studying the effect of macromolecular crowding on surface-immobilized enzymes. In another study by Zurier and Goddard, the N-terminal domain (1–60) of the Si-tag was fused to a polyethylene terephthalate (PET)-degrading enzyme (PETase), which was then immobilized in mesoporous silica nanoparticles [29]. The immobilized PETase showed improved thermal stability, leading to better PET degradation performance in unbuffered, simulated wastewater conditions. These studies demonstrate that enzyme immobilization on siliceous materials via the shortened versions of the Si-tag can be a simple and effective way to develop highly stabilized biocatalysts for environmental bioremediation.

Similarly to the L2NC, the rplB protein, a homolog of the Si-tag originating from *Mannheimia succiniciproducens*, was redesigned by removing the central domain and linking the N-terminal domain and the C-terminal domain [30]. The resulting SBP was used to immobilize avian influenza (AI) antigen on a silica surface in a nanogap field-effect transistor biosensor without surface modification. The biosensor was successful in the electrical detection of an anti-AI antibody in a label-free manner [30].

#### 2.3.2. CotB1 Derivatives

CotB1 is a spore coat protein from *Bacillus cereus* with 171 amino acids involved in biosilicification in and around the spore coat layer [31]. The zwitterionic C-terminal region (142–171) of CotB1 is essential for the *Bacillus* biosilicification [31], of which the 14 amino acid-length cationic peptide (CotB1p) at the outermost C-terminus showed a strong affinity for silica particles [17]. CotB1 and CotB1p, with a *K*_d_ of 2.09 nM and 1.24 nM at pH 8.0, respectively, were employed as fusion tags for the affinity purification of target proteins. Furthermore, a half-length CotB1p (SB7 tag) was successfully developed as an affinity tag for silica [21]. However, unlike the Si-tag, the binding of CotB1 derivatives was inhibited at high ionic strength, implying that an ionic interaction between the negatively charged silica surface and the cationic residues at the C-terminal region of CotB1 is the primary driving force for the strong binding.

Müller et al. tried to immobilize an engineered hydrolase on silica materials via the fusion of CotB1p peptide for the efficient enzymatic inactivation of the antibiotic Florfenicol. However, tagging with CotB1p resulted in a low yield of the target enzyme and severely impaired the growth of *E. coli* host cells [32]. On the other hand, tagging with the Si-tag, despite the large size, did not impair cell growth or protein expression [32].

### 2.4. Artificially Engineered SBPs

#### 2.4.1. Z_basic2_

The Z_basic2_ protein is an engineered arginine-rich variant of the Z domain, a 7 kDa three-helix bundle derived from the B domain of *Staphylococcus aureus* protein A [33]. Originally, Z_basic2_ was developed as a general purification tag for ion exchange chromatography, but it was later found that Z_basic2_ can also tightly bind to silica surfaces. The binding force of Z_basic2_ to silica is affected by pH and ionic strength, suggesting a principal role of ionic interaction in the strong binding. An enzyme fused with Z_basic2_ can be directly immobilized on porous glass from a cleared lysate with excellent binding selectivity and a high loading capacity (>30 mg protein/g support) [22]. The binding is thought to be mediated by a positively charged surface patch located across two helices of Z_basic2_, which allows for an oriented immobilization of the Z_basic2_-tethered enzyme with the full retention of biological activity [22]. Liu et al. achieved site-specific co-immobilization of two enzymes (amine dehydrogenase and glucose dehydrogenase) fused with Z_basic2_ onto mesoporous silica nanoflowers directly from the cell lysate in a one-pot process [34]. The dual-enzyme nanoreactor was self-sufficient and recyclable for chiral amine synthesis.

#### 2.4.2. Car9 Peptide

The Car9 peptide was identified from disulfide-constrained, random 12 mer peptide libraries constructed using an *E. coli* flagellar display system as a binder to graphite materials [16]. Later, it was shown to bind to silica with a high affinity. Both the disulfide-constrained form (i.e., loop form) and the linear form of Car9 were effective for silica binding. Although the *K*_d_ (~1 μM) of Car9 seems relatively high compared to those of other strong silica binders, the binding of Car9 to silica was not disrupted even by applying high ionic strength (5 M NaCl). This suggests that similar to the case of the Si-tag, strong binding involves not only an ionic interaction but also a hydrophobic interaction between the phenylalanine residues and siloxane groups. Indeed, free lysine and arginine were effective for competitively releasing Car9 from silica because their side chains have both cationic and hydrophobic characteristics. Car9 exhibits a high affinity for silica beads through the above two types of interactions and enables the efficient capture of Car9-bound proteins [16].

The Car9 tag could be added to the N- or C-terminus of a target protein, and the loading of the Car9-tagged protein was enhanced when using small-sized silica gel with large pores as the binding matrix [35]. Using an optimized small-scale purification kit, Car9-tagged proteins could be recovered from the silica matrix with high purity at a low cost within minutes [35]. This technology was extended to using silica spin columns and 96-well borosilicate plates as cheap binding matrices [36].

#### 2.4.3. Poly(Amino Acid)

A poly(amino acid) is a short peptide composed of a single type of amino acid. An example of a poly(amino acid) is the nona-arginine (Arg_9_) tag, which was originally known as a protein transduction domain for the cellular uptake of small-molecule drugs or proteins [37]. This versatile tag can also be used as a purification tag for cation exchange chromatography and an SBP for enzyme immobilization onto glass slides and silica resins [23]. However, the Arg_9_ tag is not frequently used in protein engineering because it may affect the tertiary structure of a protein [38]. Although the Arg_9_ tag at the C-terminus of a target protein can be removed by treating carboxypeptidase B that digests C-terminal arginine and lysine residues, the cleavage yield and specificity are generally low [38].

Polyhistidine-containing motifs play a critical role in silica binding (and silica synthesis also; see below), as frequently found in SBPs [12,39]. The imidazole groups form hydrogen bonds with silanol and siloxide groups on silica surfaces [15]. Indeed, the His_6_ tag, the most widely used tag for protein purification by immobilized metal affinity chromatography (IMAC), displays an affinity to silica particles [24]. A His_6_-tagged EGFP protein could be purified from *E. coli* lysate with a purity of up to 96% using silica as a solid-phase matrix, while the untagged counterpart could not bind to the matrices. Double tagging with N-terminal His_6_ and C-terminal SBPs such as Car9 or CotB1p improved the binding strength [7,24], which would be advantageous for enzyme immobilization. The binding improvement was achieved by the synergetic contribution of His_6_ and SBPs [7].

#### 2.4.4. Linker Peptide

The four repeating linker peptide (VKTQATSREEPPRLPSKHRPG)_4_VKTQTAS (4 × L) was discovered as a zeolite (aluminosilicate minerals)-binding peptide through *E. coli* cell surface display, and this cationic peptide was later found to be a strong binder toward other silica-containing materials [25]. The 4 × L fused to *Streptococcus* protein G was immobilized onto a silica surface with a vertical orientation, showing a *K*_d_ of 34.77 nM [8]. A binding test with a series of shorter derivatives showed that the 3 × L was the minimally repeating peptide capable of complete binding to zeolite or silica. Similarly to the Si-tag, more than 76% of the residues in the linker peptide tend to promote structural disorder, conferring the structural flexibility and plasticity required for the strong binding to silica by conformational adaptation [25].

In a recent study, the 4 × L peptide was fused to a thermostable β-glucosidase, an enzyme used to biotransform icariin to baohuoside I, a flavonoid compound with various pharmacological activities [40]. The fusion protein was directly purified from the cell lysate and immobilized onto Na-Y zeolite in a single step. The immobilized enzyme showed a catalytic efficiency 61% higher than that of the free enzyme, and the stability was also improved at high temperatures and in organic solvents. In another study, Care et al. utilized the 4 × L peptide for the immobilization of three thermostable hemicellulases (β-glucosidase, β-xylanase, and β-mannanase) onto Na-Y zeolite [41]. All three enzymes could be immobilized with the simultaneous formation of cross-linked enzyme aggregate (CLEA), making a stabilized, multiple-enzyme biocatalytic module for the hydrolysis of various hemicellulosic polysaccharides.

## 3. Silica-Forming Proteins/Peptides (SFPs)

### 3.1. How SFPs Work

**Figure 5:**
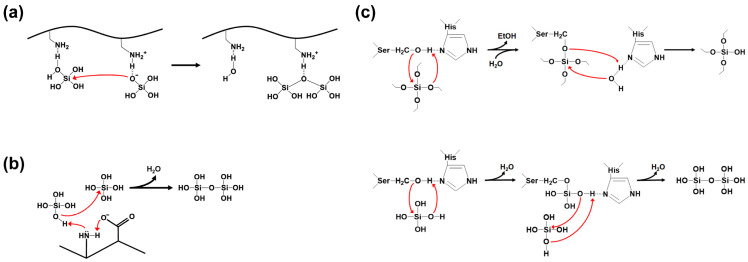
Silica polycondensation by silica-forming peptides/proteins. Red arrow indicates nucleophilic attack. (**a**) The formation of reactive silanolate by deprotonated amino group and the polycondensation through nucleophilic substitution reaction between silicic acid molecules (redrawn from reference [43]). (**b**) The charge relay effect between the amino and the carboxyl groups on adjacent amino acids promotes the activation of the lone-pair electrons on the amine nitrogen (redrawn from reference [44]). (**c**) The hydrolysis of silicon alkoxide (upper, redrawn from reference [45]) and the polycondensation of orthosilicic acids (lower, redrawn from reference [46]) catalyzed by silicatein-α.

Silica synthesis involves the process of polycondensation (polymerization) of silicic acid precursors. Silica polycondensation by SFPs is catalyzed by multiple cationic side chains on the SFPs [42,43]. Positively charged residues act as acid-base catalysts that promote the formation of siloxane bonds (Figure 5a). A deprotonated amine accepts a proton from a silicic acid to help form a reactive silanolate. A water molecule is released from a second silicic acid by a nucleophilic attack of the silanolate on the second silicic acid, facilitated by a nearby protonated amine group as a proton donor. In addition, a positively charged residue can be more polarized and activated by a closely located, negatively charged residue, leading to an improved silicic acid polycondensation by the charge relay effect (Figure 5b) [44]. This effect is particularly pronounced when the basic and acidic residues are alternatingly positioned.

In most cases, orthosilicic acid, obtained by the hydrolysis of silicon alkoxides such as tetramethyl orthosilicate (TMOS) and tetraethyl orthosilicate (TEOS), is used as the precursor for the silica synthesis by SFPs. However, some SFPs can directly utilize silicon alkoxides as the precursor. Silicatein-α is the most well-known example of this type of SFP [47]. The ‘enzyme’ silicatein-α can catalyze both the hydrolysis of silicon alkoxide molecules and the polycondensation of the resulting silicic acids (Figure 5c). The active site residues Ser26 and His165 in the silicatein-α play crucial roles in both reactions. The activity of silicatein is abolished upon thermal denaturation, suggesting its dependence on the protein’s native three-dimensional structure. The biomimetic synthesis of silica directly from TEOS was also demonstrated using synthetic block copolypeptides [48], implying that both catalytic functionalities might be implemented on a relatively small peptide that does not have a defined three-dimensional structure. The proposed mechanisms of silicatein actions can be found elsewhere [45,46].

The catalytic acceleration of silicic acid condensation is insufficient for synthesizing particulate silica, and the precipitation (flocculation) process is required to obtain silica particles. The same SFPs can also serve as flocculating agents by providing plentiful cationic and hydroxyl-containing residues that interact with the anionic surface of small oligomeric/colloidal silica via ionic interactions and hydrogen bonds [49,50,51]. During this process, SFPs are consumed and become entrapped within the synthesized silica particles. This consequence forms the basis of enzyme immobilization in biomimetic silica using SFPs. The amount of silica synthesis according to the amount of SFP shows a sigmoidal (cooperative) or a hyperbolic saturation curve [52,53,54]. The amino acid sequences and the pI values of various SFPs are summarized in Table 2.

### 3.2. Production of Enzyme and One-Step SFP-Mediated Enzyme Immobilization

**Figure 6:**
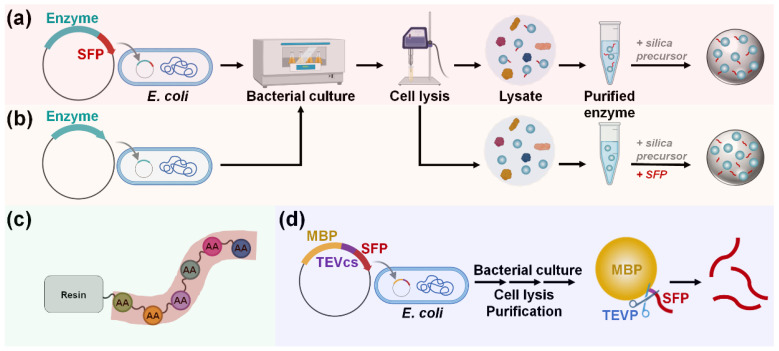
Production and immobilization of (**a**) SFP-fused or (**b**) untagged enzyme. To immobilize untagged enzymes, separate preparation and blending of sole SFP are required to induce silica coprecipitation. Sole SFP can be produced via (**c**) chemical synthesis or (**d**) recombinant expression. Abbreviations: MBP, maltose-binding protein; SFP, silica-forming peptide; TEVP, tobacco etch virus protease; TEVcs, TEVP cleavage site.

For SFP-mediated immobilization, enzymes are typically produced as fusion proteins tagged with an SFP (Figure 6a). In contrast to SBP-mediated immobilization, SFP-fused enzymes must be purified before immobilization. This is because SFP-mediated encapsulation is non-selective and requires a high concentration of SFP for efficient immobilization, making the use of cell lysate unsuitable. One-step enzyme encapsulation, which occurs simultaneously with silica synthesis, is then performed by mixing the purified enzyme solution with the silica precursors under ambient aqueous conditions. Non-recombinant enzymes or recombinant enzymes without an SFP can also be immobilized using the coprecipitation method, where enzymes are blended with separately prepared SFPs (Figure 6b). Most studies have utilized chemical peptide synthesis for the preparation of SFPs (Figure 6c). However, a recent study introduced a recombinant production method for SFPs, offering a more environmentally friendly and cost-effective approach [62]. In this method, maltose-binding protein (MBP)–SFP fusion protein, along with a flanked tobacco etch virus (TEV) cleavage site, is constructed and expressed, allowing for the SFPs to be obtained separately following treatment with TEV protease (Figure 6d). The genetic fusion of an SFP to a target enzyme provides benefits over the coprecipitation method as the peptide can be easily produced alongside the enzyme at a low cost, and a higher immobilization yield can be attained, particularly when the concentration of the SFP-fused enzyme is high [63].

### 3.3. Naturally Occurring SFPs

#### 3.3.1. Silaffin-Derived R5 Peptide

Silaffin is a family of cell wall peptides originating from the diatom *Cylindrotheca fusiformis* and is involved in biosilica formation in diatom cell walls in vivo [50]. The representative silaffin, silaffin-1A_1_ (SSKKSGSYSGSKGSK), is one of the in vivo endoproteolytic products of the silaffin precursor polypeptide sil1p. The polypeptide backbone of silaffin-1A_1_ is highly cationic due to the high Lys content and the lack of acidic residues. However, the extensive posttranslational modifications with long-chain polyamines (on Lys residues) and phosphates (on Ser residues) eventually render the native silaffin-1A_1_ uniquely zwitterionic, allowing for the supramolecular assembly of the peptides via electrostatic interactions as an essential prerequisite for silica formation [64,65].

The R5 peptide (SSKKSGSYSGSKGSKRRIL) is one of the repeat sequences of the sil1p [50]. Unlike the native silaffin-1A_1_ capable of precipitating silica under a wide range of pH conditions, the synthetic R5 peptide shows silica precipitation activity only at pH > 6 due to a lack of long-chain polyamine modifications [50,53]. Since the first demonstration of enzyme immobilization in biomimetic silica support [66], the R5 peptide has undoubtedly been the most widely used SFP due to its high efficiency in silica synthesis despite its relatively short length [5,67].

In a recent study, by using the R5-fused bovine CA (bCA), it was shown that the addition of metal cations such as Na^+^ and Cs^+^ during the immobilization increased the silica synthesis with high packing density, leading to an 11- to 18-fold increase in the stability of the immobilized bCA-R5 compared to the counterpart without the addition of the cation [68]. Whether the cation addition was effective for the further stabilization of the R5-fused enzyme was determined by both the pH condition and the enzyme’s surface electrostatic nature, suggesting unique interactions among all the components (cation, silica, and R5-fused enzyme) during the immobilization reaction. In another study, Lee et al. fabricated an ultrathin (~1.5 nm) and mesoporous silica layer on triblock copolymer-based micelle nanoparticles that were functionalized with the R5 peptide and a CA enzyme [69]. The synthesis of the silica shell was performed under a TMOS–water biphasic system. The immobilized enzyme was highly stabilized and showed no mass transfer limitation for the catalytic activity.

The R5-mediated biomimetic silicification has also been employed to stabilize integral membrane proteins (IMPs). These proteins are highly prone to aggregation and unstable in solution, impeding further studies and handling. Designed amphiphilic β-strand peptides, such as BP-1, can associate with IMPs and stabilize them in a detergent-free buffer [70]. Bialas and Becker used an R5-fused BP-1 to further stabilize diacylglycerol kinase (DGK), an IMP [70]. The stability of encapsulated DGK was improved against protease attack and extremely low pH.

#### 3.3.2. EctP Peptides

Yeo et al. performed the BLAST search using the silaffin R5 peptide sequence as a query and identified SFP candidates (EctP1 and EctP2) from the brown algae *Ectocarpus siliculosus* [55]. The EctP peptides exhibited better abilities for silica synthesis than the R5 peptide; particularly, EctP1 was superior at the relatively low pH of 6, at which the R5 peptide rarely showed silicification activity.

#### 3.3.3. Histidine-Rich Kpt Peptide and Glassin

Inspired by His-rich peptides screened from the phage display library [12], Nguyen et al. performed the BLAST search using the Si3-8 (Kps peptide) sequence as a query. As a potential SFP, the Kpt peptide (KPTHHHHHHDG) was discovered from the marine bacterium *Ruegeria pomeroyi* DSS-3 [56]. Compared to the R5 peptide, the Kpt peptide performed better at the relatively high pH of 8. Obviously, the natural Kpt sequence contains the His_6_ sequence, which gives the peptide dual functionalities as both an SFP and a purification tag.

Another example of a His-rich protein is glassin, a 23 kDa protein discovered in the marine sponge *Euplectella aspergillum* [71]. When hydrolyzed TMOS was used as the silica precursor, the glassin showed silica-precipitating activity even after heat denaturation. The silicification activity was attributed to the histidine and threonine-rich HT domain [72]. However, this activity has not been exploited for enzyme encapsulation in bioinspired silica.

#### 3.3.4. Lysozyme

Lysozyme is an antibacterial enzyme that hydrolyzes and degrades bacterial cell walls, leading to the lytic death of bacterial cells. As a highly basic protein containing a high content of hydroxyl group, lysozyme can direct the formation of silica along with the encapsulation of the active lysozyme to create highly stabilized, antibacterial nanocomposite materials [57]. The immobilized lysozyme was shown to be firmly trapped inside the silica by steric effects [73]. Although it is currently uncertain how the entrapped enzyme could physically access the bacterial cell wall components for the lytic action, a detailed structural study on the lysozyme–silica composite suggested that the presence of surface-accessible lysozyme was responsible for the observed antibacterial activity [74]. Because additional enzymes can be simultaneously immobilized during the silica synthesis, lysozyme-mediated silica synthesis offers an economical and efficient method for fabricating versatile nanocomposites equipped with antibacterial activity.

#### 3.3.5. Protamine

Protamines are a group of small DNA-binding peptides found in sperms, contributing to the condensation and stabilization of the spermatid genome [75]. Notably, protamines are strongly cationic with high contents of Arg, suggesting possible use as SFPs. For example, salmon protamine comprises 32 residues (excluding the first Met), of which 21 (66%) are Arg. Salmon protamine was first used as an SFP to fabricate an alginate/protamine/silica hybrid capsule [58]. The enzyme β-glucuronidase was pre-encapsulated within the liquid-core alginate, and then the anionic surface was coated with the cationic protamine by which biomimetic microscale silica shell was subsequently formed from sodium silicate solution. The silica shell effectively prevented the shrinking or swelling of the core alginate capsule, and the catalytic activity and recycling stability of the encapsulated enzyme were significantly improved after the biomimetic silicification. In a subsequent study, biomimetic layer-by-layer mineralization was demonstrated using the protamine for biocatalytic nanoscale coatings of silica or titania [76]. Protamine-conjugated glucose oxidase was stably incorporated into the desired layer, exhibiting higher catalytic activity when located closer to the outer surface. Although protamine was successfully used as an effective SFP, enzyme encapsulation in a genetically fused form has not yet been proven.

#### 3.3.6. Arginine-Rich Peptides

In addition to protamines, other arginine-rich SFPs have been discovered. The Salp1 peptide was derived from a siliceous choanoflagellate [59]. Unlike the R5 peptide, a wet silica gel was fabricated when using Salp1 unless the ionic strength of the silicifying solution sufficiently increased. Notably, the release of the entrapped GFP-Salp1 from the silica matrix was much slower than the GFP-R5. This was attributed to two Cys residues in Salp1 that can form intermolecular disulfide bonds, resulting in a more stable encapsulation. The different release profiles of different SFPs might be exploited for the controlled release of multiple proteins, e.g., in a drug delivery system.

Min et al. found multiple arginine-rich peptides, all showing silicifying activity [52]. Among them, the 22-residue RSGH peptide, which has the duplicated sequence from the 11-residue peptide from *Equus caballus*, was a more efficient SFP than the R5 peptide under acidic (pH 5 and pH 6) conditions [52]. Similarly, the 23-residue Wa-RSG peptide from *Winogradskyella arenosi* worked as well at pH 6 as it did at pH 7 [60]. These peptides were adsorbed onto the surface of yeast or bacterial cells, respectively, and the cells were encapsulated in silica shells, resulting in improved tolerance to dehydration and UV-C irradiation [52,60].

#### 3.3.7. Silicatein

Silicatein filaments were first discovered in silica spicules of the marine sponge *Tethya aurantia*. The filaments consist of three different silicatein subunits designated as α, β, and γ, of which silicatein-α comprises 70% of the mass of the filaments. Unlike the abovementioned other SFPs, the silicateins were found to be able to catalyze silica polymerization directly from the non-hydrolyzed, silicon alkoxide precursors TEOS or TMOS at neutral pH [45,47], as described in Section 3.1. Silicatein-α has not been used widely as an SFP for enzyme immobilization because of the relatively large size (23.3 kDa), low expression level in bacterial hosts, and inherent poor solubility [77]. Nonetheless, a recent study showed that interfacial silica was formed on chitosan gel using silicatein fused with a solubility-enhancing protein and a chitin-binding domain, and the stability and reusability of horseradish peroxidase encapsulated in the chitosan gel were improved [78].

### 3.4. Artificially Engineered SFPs

#### 3.4.1. Synthetic Peptides from Phage Display Biopanning

Among approximately 10^9^ random dodecapeptides from a combinatorial M13 phage display library, silica-binding peptides were screened via biopanning on amorphous silica [12]. Several peptides capable of silica binding were selected after multiple rounds of panning, among which His-rich Si4-1 and Arg-rich Si4-10 peptides had particularly strong binding affinity to silica surfaces. Interestingly, some of the selected SBPs also showed silica-precipitating activity, and the highest activity was observed when using the strongest SBP, Si4-1. Notably, no silica precipitate was visible when the Si4-10 was used, implying that silica-binding ability is not a sufficient requisite for silica-forming activity. Despite the short length (12-mer), these peptides have not been used as SFPs for enzyme encapsulation due to the relatively weak silica-forming activity compared to that of the R5 peptide. Instead, studies were reported where the Si4-1 [79,80] and the Si4-10 [81] were used as SBPs for enzyme immobilization.

#### 3.4.2. Elastin-like Polypeptide

Elastin-like polypeptides (ELPs) are nature-inspired, artificial polypeptides with a repeated sequence of VPGXG motif, where the “X” is any amino acid except for proline [82]. ELPs undergo a thermally responsive, reversible phase separation, making them attractive materials for protein purification and nanoassembly. Previously, ELPs fused with the R5 peptide were constructed to fabricate hybrid nanoparticles with the self-assembled core ELP micelles coated by silica shells [82]. Later, Lin et al. endowed an ELP with the silica-forming ability by incorporating Lys, Val, and Phe at the “X” sites of the 40 repeated pentapeptides in the ratio of 1:8:1 [61]. The resulting cationic ELP[KV_8_F-40] as an SFP was then fused with the enzyme xylanase, and the fusion protein was highly purified by the inverse transition cycling method. The self-assembled xylanase-ELP[KV_8_F-40] was efficiently encapsulated within a biomimetic silica matrix, showing a high immobilization yield and improved stability. In other studies by the same group, ELP[KV_8_F-40] was also utilized for the immobilization of a cutinase in silica or magnetic–silica nanoparticles toward enhanced PET biodegradation with improved thermal stability and reusability [83,84].

## 4. Future Perspectives

While SBPs allow for a facile and oriented immobilization of enzymes on the surface of silica and, thus, a high retention of enzymatic activity after immobilization, surface-exposed enzymes are generally susceptible to external physical and chemical stresses. In contrast, SFP-mediated silica encapsulation provides a more stable niche for enzymes, despite the inability to control enzyme orientation. The reduction in enzymatic activity due to mass transfer limitations following encapsulation is another limitation, which can be alleviated by reducing silica thickness and/or controlling silica morphology to achieve a high surface-to-volume ratio. To this end, achieving sub-nanoscale control of silica synthesis is essential, potentially through the combination of intricately designed SFPs and other additives that influence the silica synthesis process [51,69]. Structure-guided in silico approaches, such as molecular dynamics simulations, are becoming essential for studying and predicting silica synthesis by SFPs [65], as well as the orientation, binding strength, and conformational changes in enzymes upon immobilization [85,86,87,88]. When combined with advanced experimental techniques, these computational methods could ultimately lead to the elucidation of design principles for SFPs and the silica synthesis process. Such advances would facilitate the development of methods for the oriented and ordered encapsulation of enzymes within silica, thereby further enhancing enzymatic activity without compromising the benefit of increased stability.

For successful industrial applications, it is essential to address the large-scale and cost-effective manufacturing of tailor-made biocatalytic silica using the bioinspired approach utilizing SFPs [89]. It is also important to note that silica is susceptible to dissolution by various external factors, such as high pH, which can restrict its long-term use in harsh industrial environments [90]. This dissolution may be effectively reduced by applying passivation layers to silica particles [90]. Interestingly, diatomite, a form of diatom-derived biosilica, shows resistance to dissolution even at a pH as high as 11, presenting a cost-effective, environmentally friendly, and stable alternative support to which SBP-fused enzymes can bind [20,91].

From a materials perspective, silica-based hybrid materials offer advantages over bare silica, such as more suitable surface properties for enzyme immobilization and additional useful functionalities [84,92,93]. For instance, silica-coated magnetic nanoparticles facilitate the rapid magnetic separation and recovery of immobilized enzymes [84]. In this context, the further exploration of more compatible silica-binding and silica-forming methods for use on silica hybrid materials will be necessary.

## 5. Conclusions

In this review, various SBPs and SFPs, categorized into naturally occurring and artificially engineered types, are summarized, providing customizable options for enzyme immobilization on siliceous materials under different optimal conditions. The proposed mechanisms and recombinant production of SBPs and SFPs are also discussed. Various case studies demonstrate the practical applications of these SBPs and SFPs in enzyme immobilization. Although SBPs and SFPs share some common features in their sequences and amino acid compositions, the specific determinants that confer silica-forming ability have barely been elucidated. Expanding our understanding of the underlying mechanisms of silica binding and formation will lead to designing new SBPs and SFPs that are more efficient and controllable, thereby broadening their functionalities and applications.

## Figures and Tables

**Table 1:** List of silica-binding peptides (SBPs).

Name	Amino Acid Sequence	Length	pI ^a^	Reference
Naturally occurring SBP				
Si-tag	MAVVKCKPTSPGRRHVVKVVNPELHKGKPFAPLLEKNSKSGGRNNNGRITTRHIGGGHKQAYRIVDFKRNKDGIPAVVERLEYDPNRSANIALVLYKDGERRYILAPKGLKAGDQIQSGVDAAIKPGNTLPMRNIPVGSTVHNVEMKPGKGGQLARSAGTYVQIVARDGAYVTLRLRSGEMRKVEADCRATLGEVGNAEHMLRVLGKAGAARWRGVRPTVRGTAMNPVDHPHGGGEGRNFGKHPVTPWGVQTKGKKTRSNKRTDKFIVRRRSK	273	10.9	[19]
L2NC	MAVVKCKPTSPGRRHVVKVVNPELHKGKPFAPLLEKNSKSGGRNNNGRITTRHIGGGHKQRVLGKAGAARWRGVRPTVRGTAMNPVDHPHGGGEGRNFGKHPVTPWGVQTKGKKTRSNKRTDKFIVRRRSK	131	12.1	[19,20]
CotB1p	SGRARAQRQSSRGR	14	12.6	[17]
SB7	RQSSRGR	7	12.3	[21]
Artificially engineered SBP				
Z_basic2_	VDNKFNKERRRARREIRHLPNLNREQRRAFIRSLRDDPSQSANLLAEAKKLNDAQPK	57	11.4	[22]
Car9	DSARGFKKPGKR	9	11.1	[16]
Arg_9_	RRRRRRRRR	9	12.9	[23]
His_6_	HHHHHH	6	7.2	[7,24]
Linker peptide	VKTQATSREEPPRLPSKHRPGVKTQATSREEPPRLPSKHRPGVKTQATSREEPPRLPSKHRPGVKTQATSREEPPRLPSKHRPGVKTQTAS	91	11.6	[25]

^a^ The pI values were calculated at the Expasy server (https://web.expasy.org/compute_pi/ (accessed on 3 September 2024)) based on the indicated amino acid sequences.

**Table 2:** List of silica-forming peptides (SFPs).

Name	Amino Acid Sequence	Length	pI ^a^	Reference
Naturally occurring SFP				
R5	SSKKSGSYSGSKGSKRRIL	19	11.2	[50]
EctP1	SSRSSSHRRHDHHDHRRGS	19	11.8	[55]
EctP2	SSKKSGERHHRSA	13	11.0	[55]
Kpt	KPTHHHHHHDG	11	7.2	[56]
Lysozyme	KVFGRCELAAAMKRHGLDNYRGYSLGNWVCAAKFESNFNTQATNRNTDGSTDYGILQINSRWWCNDGRTPGSRNLCNIPCSALLSSDITASVNCAKKIVSDGNGMNAWVAWRNRCKGTDVQAWIRGCRL	129	9.3	[57]
Protamine	MPRRRRSSSRPVRRRRRPRVSRRRRRRGGRRRR	33	13.3	[58]
Salp1	CGRRRGGRGGRGRGGCGRRR	20	12.3	[59]
RSGH	RRSGHSHEGRRRRSGHSHEGRR	22	12.2	[52]
Wa-RSG	RRSSGGSRKRDDKPRGDRRSSGG	23	11.9	[60]
Silicatein-α	AYPETVDWRTKGAVTGIKSQGDCGASYAFSAMGALEGINALATGKLTYLSEQNIIDCSVPYGNHGCKGGNMYVAFLYVVANEGVDDGGSYPFRGKQSSCTYQEQYRGASMSGSVQINSGSESDLEAAVANVGPVAVAIDGESNAFRFYYSGVYDSSRCSSSSLNHAMVITGYGISNNQEYWLAKNSWGENWGELGYVKMARNKYNQCGIASDASYPTL	218	4.9	[47]
Artificially engineered SFP				
Si4-1	MSPHPHPRHHHT	12	9.6	[12]
ELP[KV_8_F-40]	VPGKGVPGVGVPGVGVPGVGVPGVGVPGVGVPGVGVPGVGVPGVGVPGFGVPGKGVPGVGVPGVGVPGVGVPGVGVPGVGVPGVGVPGVGVPGVGVPGFGVPGKGVPGVGVPGVGVPGVGVPGVGVPGVGVPGVGVPGVGVPGVGVPGFGVPGKGVPGVGVPGVGVPGVGVPGVGVPGVGVPGVGVPGVGVPGVGVPGFG	200	10.5	[61]

^a^ The pI values were calculated at the Expasy server (https://web.expasy.org/compute_pi/ (accessed on 3 September 2024)) based on the indicated amino acid sequences.

## Data Availability

Not applicable.
